# Antioxidant and antimicrobial activity of two Costa Rican cultivars of ber (*Ziziphus mauritiana*): An underexploited crop in the American tropic

**DOI:** 10.1002/fsn3.3317

**Published:** 2023-03-17

**Authors:** Eric Cubero‐Román, Yendry Carvajal‐Miranda, Gerardo Rodríguez, Victor Álvarez‐Valverde, Pablo Jiménez‐Bonilla

**Affiliations:** ^1^ Laboratorio de Fitoquímica, Escuela de Química Universidad Nacional Heredia Costa Rica; ^2^ Agricultural Chemistry Department University of California Davis Davis California USA; ^3^ Laboratorio de Productos Naturales y Ensayos Biológicos, Escuela de Química Universidad Nacional Heredia Costa Rica; ^4^ Cátedra de Ciencias Químicas Universidad Estatal a Distancia San José Costa Rica

**Keywords:** antimicrobial, Ber, nutraceutical, polyphenol, proanthocyanidin, *Ziziphus mauritiana*

## Abstract

Ber is an antioxidant‐rich fruit from Asia and has recently been cultured in Central America. The antioxidant capacity and antimicrobial activity of *Z. mauritiana* cultured of bers from Guanacaste, Costa Rica, were evaluated. Two farm locations and two cultivars were evaluated. Total polyphenolic compounds (TPC), proanthocyanidin compounds (PAC), and ascorbic acid were spectrophotometrically quantified. Antioxidant activity has been analyzed using the DPPH method. Antimicrobial susceptibility was determined using the Kirby‐Bauer disk diffusion method. Ber samples contained 11–44 mg GAE/g TPC. Green fruits and leaves had the highest concentrations. The ascorbic acid concentration in ber fruits was determined between 251 and 466 mg/100 g. Ber vitamin C content is higher than most common fruits. Proanthocyanidin compounds were determined between 1.8 and 9.9 mg 4‐MCG/g, and the highest concentration was observed in the leaves. Our samples showed the antioxidant activity of 90–387 μmol TE/g, which was moderate activity. The nutritional quality of ber fruits was related to maturity conditions. The ber fruits, a crop from Asia previously adapted to live in Costa Rica, are rich in vitamin C and TPC, and the concentration of those metabolites was even higher than the concentration reported in bers grown in other countries. The TPC and PACs had an interestingly wide antimicrobial spectrum. Cultivars and farm locations have a significant effect on metabolite production.

## Graphical abstract

Ber has been cultivated in Asia for many years, and it has been introduced to the American tropic in recent years. We studied the characteristics and benefits of the cultivation of this fruit in Central America. We found out it produces higher antioxidant levels than many other reports, and also shows antimicrobial properties.

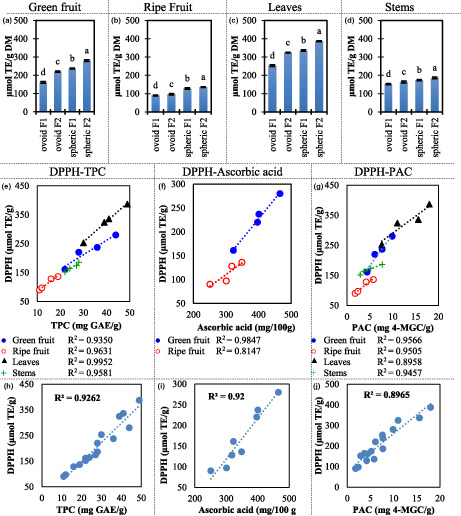

## 1 INTRODUCTION

*Ziziphus mauritiana* is a fruit native to the subcontinent of India and distributed in some regions of Africa, Oceania, and Asia, such as India, Afghanistan, Algeria, Egypt, Kenya, Pakistan, Malaysia, southern Africa, Japan, Nepal, Australia, Philippines, and Pacific Islands (Prakash et al., 2021). It is called Ber, Indian jujube, Desert apple, Indian plum, Malay apple, Chinese apple, dunks, and Chinese date. Ber is a bush or small tree with round or elliptical sheet‐shaped leaves, green‐yellow‐red flowers, and armed branches with two thorns per node (Zamora et al., 2010).

Several bioactive compounds have been found in *Z. mauritiana*, such as the alkaloids protopine and berberine; flavonoids such as quercetin and kaempferol; sterols that are sitosterol, stigmasterol, lanosterol, and diosgenin; a complex mixture of flavonoids, polyphenolic hydrolyzable tannins; oses and holosides; mucilage, sterol, triterpenoids, cardiac glucosides, and leucoanthocyanidins; terpenes such as some saponins; and others (Prakash et al., 2021). Secondary metabolites from *Z. mauritiana* have been found active as antidiarrheal (Dahiru et al., 2006), antibacterial (Abalaka et al., 2010), poison antidote for carbon tetrachloride damage in the liver (Dahiru et al., 2005), antioxidant (Zozio, Servent, Cazal, et al., 2014), antihemorrhagic, anticancer, antidiabetic, and sleep booster (Zhang et al., 2020).

China holds 90% of the world's production, mostly for domestic consumption, and its market represented 16.5 million tons in 2019 (Zhang et al., 2020). Besides its popularity in Asia and Africa, this crop is not very well known in Central America and the American tropics. Although it is considered a crop from temperate regions, *Z. mauritana* has been introduced in Costa Rica during the late stage of the twentieth century. In 1993, around 20 ha were cultivated (MAG‐INTA, 2022); most of them were in Nandayure, Guanacaste, a place located in the dry corridor of Central America. The National Institute for Rural Development (INDER, by its Spanish acronym, formerly IDA) promoted the farming of *Z. mauritiana* and *Psidium guajava* in Paquera, Nicoya Peninsula during the early ‘00 s (INDER, 2014). Graft plants from Costa Rica and Taiwan were used to establish the cultures. Currently, there are an estimated 400 trees producing 50 kg/year each, approximately, according to local producers. *Z. mauritiana* can grow in tropical dry climate conditions, growing well adapted in the tropics, and it is an excellent alternative for agricultural production.

This study is the first phytochemical evaluation of *Z. mauritiana* in Central America, and it provides valuable insights into the potential of this fruit in terms of antioxidant and antibiotic activity, as well as the content of polyphenolic metabolites and vitamin C, when grows in the American tropic. In this work, the objective was to evaluate the antioxidant and antimicrobial activity of *Z. mauritiana* cultured in Guanacaste, Costa Rica.

## 2 METHODOLOGY

### 2.1 Plant tissues

Stems, leaves, and fruits, at two maturity conditions of *Ziziphus mauritiana*, were collected from two local farms (referred to as F1, and F2) at Cangelito, Nandayure (10°0′8′′, 85°10′48′′), Nicoya Peninsula, Costa Rica. The farms are located 2 km apart. The samples were freeze‐dried and ground to 0.5 mm. The maturity stages are defined as follows: the green fruits are full size strong green color, and ripe fruits are “bleached” green turning yellow. According to Zozio's scale, green fruit is stage 1, and ripe fruit stage 2 (Zozio, Servent, Hubert, et al., 2014). Additional information about the crops is in Figure S1. Cultivars are defined according to the fruit shape: ovoid or round shape. Composed samples were taken from random places on each farm during the dry season (December–March), containing around 30 fruits per sample. Three repetitions of each sample were used for the following analysis.

### 2.2 Polyphenol extraction

We preliminary tested four solvent mixtures: acetone:methanol:water (4:4:2), acetone:ethanol:water (4:5:1), acetone: water (7:3), and ethanol 95%. Seventy‐five milligrams of plant material was extracted three times with 3 mL of each solvent for 10 min in an ultrasonic bath. Then, the mixture was centrifuged at 1760 × g for 5 min. The three extraction supernatants were collected together and adjusted to 10 mL. Then, the samples were analyzed using the Folin‐ciocalteau method, as described hereunder, in order to determine which solvent offers better extraction rate acetone: water (7:3) yields the highest polyphenol concentration (Results shown in Figures S2 and S3). Similarly, the number of extractions were evaluated using from 1 to 5 steps of 2 mL, instead of 3 mL, as the only change to the method described above. The maximum concentration was observed with three extractions. Samples extracted at optimum conditions were used for the next steps.

### 2.3 Preparation of *Ziziphus mauritiana's* phenolic standard ( *zm*‐PS)

We purified the phenolic fraction from the same fruit to obtain a standard representative of the veritable mass of the sample. This approach has been previously validated with other proanthocyanidin extracts (Feliciano et al., 2012). Ten grams from Z. mauritiana. previously freeze‐dried and grounded, were extracted with 70 mL of acetone: water (7:3). Then, the extract was concentrated in a rotatory evaporator at 40°C and reduced pressure, and purified through column chromatography, utilizing C‐18 and distilled water, as stationary phase and eluent, respectively. The fraction containing the highest number of polyphenols was identified using Folin‐Ciocalteau's method. Then, it was freeze‐dried and used to quantify total phenolics and proanthocyanidins.

### 2.4 Total polyphenol compounds (TPC) quantification

75 mg of plant tissue, extracted as mentioned hereabove, were analyzed utilizing the Folin‐Ciocalteau method, according to our previously published protocol (Syedd‐León et al., 2020). Two standard curves of 0–0.200 g/L zm‐PS and 0–0.12 mg/L gallic acid were used.

### 2.5 Preparation of *Ziziphus mauritiana's* proanthocyanidin standard ( *zm*‐PAC)

We prepared *zm*‐PAC using a modification of the previously published protocol (Birmingham et al., 2021). Ten grams of each *Ziziphus mauritiana* sample, previously freeze‐dried and grounded, were individually extracted in acetone: water (7:3). The solid was dumped, and the extracts were concentrated by rotavaporation at 40°C and reduced pressure. Then, the concentrated extract was purified using a glass chromatographic column filled with Sephadex LH‐20 as a stationary phase. First, the column was eluted with ethanol: methanol (1:1) to eliminate impurities. Then, the mobile phase was changed to acetone: water (7:3), and the proanthocyanidin fraction was collected. Finally, the proanthocyanidin solution was dried by rotavaporation followed by lyophilization and used as *Ziziphus mauritiana*'s proanthocyanidin standard (*zm*‐PA).

### 2.6 Total proanthocyanidins (PAC) determination

We followed our previously published protocol (Mesén‐Mora et al., 2019) with minor modifications. Two microliters of a 0.1 mg/mL solution of 4‐(Dimethylamino) cinnamaldehyde (DMAC, obtained from Merck KGaA, Darmstadt, Germany), and 70 μL of each plant extract (as described above) were mixed into a microplate well. Absorbance was recorded at 640 nm against a 0–0.03 mg/L 4'‐O‐methyl‐gallocatechin standard curve. Additionally, a second standard curve of 0–0.16 g/L *zm*‐PS was utilized.

### 2.7 Ascorbic acid determination

0.4 g of fruit was extracted three times with distilled water, filtrated, and adjusted to 50 mL. Then, ascorbic acid was determined by iodine redox titration, as reported before (Satpathy et al., 2021) with minor modifications. Five milliliters of the previously prepared solution, 1 mL of 15% HCl, and 1 mL of 1% starch solution were titrated with 0.05 mol/L iodine solution, previously standardized, using sodium thiosulphate.

### 2.8 Antioxidant capacity determination

Antioxidant Capacity was determined by DPPH following our previously published protocol with some modifications (Araya et al., 2017). Thirty microliters of the previously described extracts were individually mixed in microplate wells with 270 μL of 0.042 mg/mL DPPH solution. Solutions were incubated for 20 min and Trolox equivalents (TE) were determined by recording the absorbance at 517 nm in a microplate reader BiotekSynergy HT.

### 2.9 Antibiotic activity

Antibacterial activity was tested against four bacteria: *Staphylococcus aureus* ATCC 25923, *Bacillus subtilis* ATCC 6633, *Escherichia coli* ATCC 25922, and *Pseudomonas aeruginosa* ATCC 9027. For this purpose, 250 μL of each bacterial suspension with 0.5 McFarlan turbidity (1.8 × 10^6^ UFC/mL) were individually cultured into Muller Hinton agar plates. Then, three 6 mm glass fiber disks containing 50 μL of extract, 30 μg chloramphenicol (as positive control), and 50 μL acetone: water (7:3, as negative control), respectively, were placed in different positions of each inoculated plate and incubated at 35°C during 24 h. Finally, the inhibition ring was measured.

### 2.10 Statistical analysis

Results were calculated using mean values and standard deviations (M ± SD). Post hoc Tukey's tests were performed for the analysis of TPC, PAC, vitamin C, antioxidant activity, and antibiotic activity.

## 3 ANALYSIS AND RESULTS

### 3.1 Total phenolic compounds (TPC)

**FIGURE 1:**
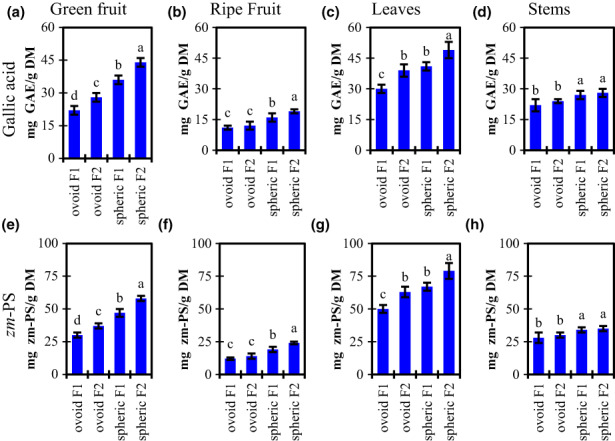
Total polyphenol content in green fruits (a, e), ripe fruits (b, f), leaves (c, g), and steams (d, h) from two cultivars of *Ziziphus mauritana*. Results are expressed in terms of gallic acid (a–d) and *Z. mauritana* polyphenol standard (*zm*‐PS, e–h). Letters on top of columns represent compact display letters of the Tuckey test. Error bars represent standard deviation.

Figure 1 summarizes the results for TPC using both Gallic Acid and the *Ziziphus mauritiana* standard *zm*‐PS. TPC concentration was between 57 and 64% higher for the green spheric cultivar, respecting the ovoid cultivar. The green ovoid cultivar has a TPC of 56%–71% higher than ripe ones. TPC concentration is higher for the spheric cultivar all over the plant. Such difference is bigger in the fruits than in other tissues. The TPC of the spheric cultivar's leaves is 26%–37% higher than the ovoid cultivar and TPC of stems is 16%–22% higher (spheric vs ovoid cultivar).

Table 1 shows the TPC reported on the literature for some ber fruits from different locations over the globe. Interestingly, the fruits included in this study had a TPC higher than most reports. For example, in Thailand and Pakistan, ber fruits had a polyphenol content of around 8.5 mg GAE/g (Ashraf et al., 2015; San et al., 2013), but we found samples reaching 44 mg GAE/g (more than 5‐fold‐up). The polyphenolic content was remarkably high in the fruits harvested in Costa Rica, respecting other latitudes.

**TABLE 1:** Comparison between polyphenolic content of Ber from different locations.

Fruit	Species	Country	TPC (mg GAE/g)	Reference
Ber seeds	*Z. mauritiana*	Thailand	8.34 ± 0.43	San et al. (2013)
Ber	*Zizyphus sp*.	India	1.72–3.29	(Koley et al. (2016)
Ber	*Z. mauritiana*	Pakistan	8.469 ± 0.092	Ashraf et al. (2015)
Ber	*Z. mauritiana*	India	2.38–4.00	Koley et al. (2011)
Ber	*Z. mauritiana*	Costa Rica	11–44	This work

Abbreviations: GAE, Gallic Acid Equivalents; TPC, Total Phenolic Content.

Also, we expressed the results of Figure 1 in terms of two kinds of standards: the gallic acid equivalents and the *Ziziphus mauritiana* polyphenol standard (*zm*‐PS). Gallic acid equivalents are the most common units utilized to describe TPC. Although, the molecular weight distribution of polyphenolic compounds in the fruits is not often represented by gallic acid. Then, we used the purified polyphenolic standard from the same plant to represent a more realistic mass. This approach had been used by other authors (Feliciano et al., 2012). There was a similar *zm*‐PS/GAE ratio for individual measurements within each part of the plant, but differences between groups. Therefore, *zm*‐PS/GAE ratio is 1.18 ± 0.07 for ripe fruits, 1.33 ± 0.03 for green fruits, 1.63 ± 0.02 for leaves, and 1.26 ± 0.01 for stems. The lowest *zm*‐PS/GAE ratio was found in ripe fruits. It means, on average, polyphenolic compounds from ripe fruits had a smaller molecular weight than those from the other parts of the plant. The *zm*‐PS/GAE ratio difference between green and ripe fruits was explained by the oxidative processes taking place during ripening. Those processes reduce the molecular weight of phenolic compounds. Also, there was a significant reduction in the TPC. For example, the spheric cultivar from field F2 reduces its TPC from 44 to 19 mg GAE/g (58 to 24 mg *zm*‐PS/g) (Figures 1a,e). The reduction of TPC during ber ripening had been reported before (Huang et al., 2017).

On the contrary, the leaves contained a higher ratio, which means, the compounds with higher molecular weight are present in the leaves. Harvest location affects TPC, being F2 values greater than F1 except for stems, whereas F1 and F2 were not significantly different.

### 3.2 Ascorbic acid content

**FIGURE 2:**
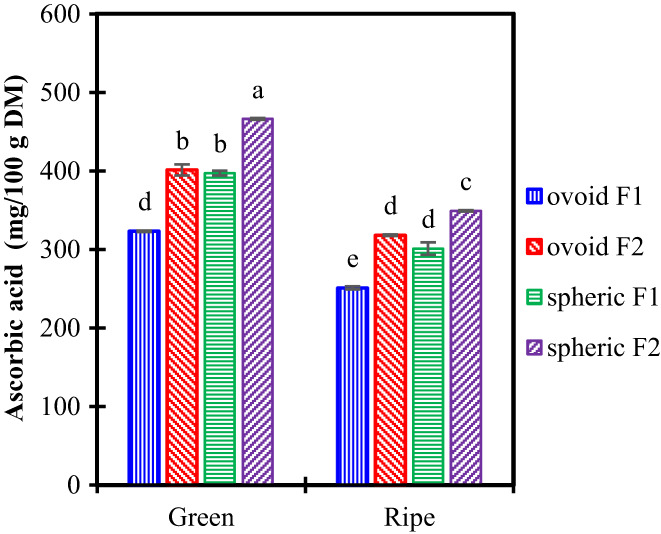
Ascorbic acid in two cultivars of ber fruits. Letters on top of columns represent compact display letters of Tuckey test. Error bars represent standard deviation.

The Vitamin C content of fruits is shown in Figure 2. The ascorbic acid accounted for 323–466 mg/100 g in the green fruit and dropped to 251–349 mg/100 g in the ripe fruit. It was consistent with previous reports from 46 cultivars of New Mexico and 121 cultivars from China, reporting an ascorbic acid content in the range of 225 to 820 mg/100 g (Huang et al., 2017).

For each cultivar and harvest location, the ascorbic acid concentration decreased by 20%–25% during the ripening process. According to Zozio et al. Zozio, Servent, Cazal, et al. (2014) and Zozio, Servent, Hubert, et al. (2014), ascorbic acid concentration increased with sucrose concentration until a maximum value was reached. Then, both sucrose and ascorbic acid decreased. Hasan et al. (2022) reported similar behavior. It probably means that the fruits included in this study had been harvested at or after the maximum ascorbic acid concentration.

Table 2 describes several fruits considered rich in Vitamin C. Ber had an ascorbic acid content higher than many of those rich‐vitamin C fruits, such as pineapple, kiwi, avocado, lemon, or guava. Ber concentration was approximately 1 to 7 fold‐up the concentration of the fruits mentioned above.

**TABLE 2:** Ascorbic acid content in several vitamin C‐rich fruits.

Fruit	Species	Ascorbic acid (mg/100 g)	Reference
Pineapple	*Ananas comosus*	61.0 ± 0.1	Valente et al. (2011)
Kiwi	*Actinidia deliciosa*	91.0 ± 0.2	Valente et al. (2011)
Avocato	*Persea americana*	160 ± 1	Shyla and Nagendrappa (2013)
Lemon	*Citrus x limón*	177 ± 1	Shyla and Nagendrappa (2013)
Guava	*Psidium guajava*	198 ± 2	Valente et al. (2011)
Amla	*Emblica officinalis*	385 ± 1	Valente et al. (2011)
Chinese jujube	*Ziziphus jujuba*	387–555 ± 50	Wojdyło et al. (2016)
Ber	*Ziziphus mauritiana*	251–466 ± 8	This work

### 3.3 Total proanthocyanidin (PAC) concentration

**FIGURE 3:**
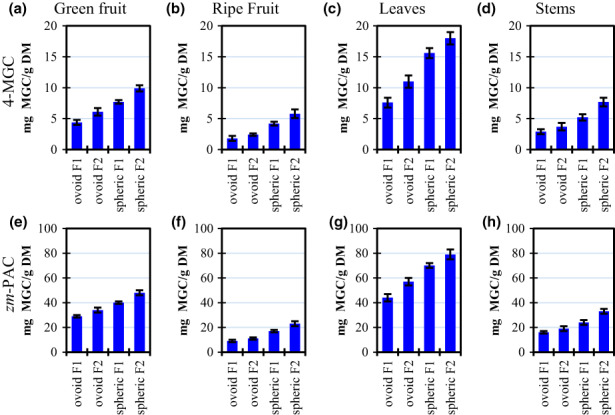
Total proanthocyanidins (PAC) in green fruits (a, e), ripe fruits (b, f), leaves (c, g), and steams (d, h) from two cultivars of *Ziziphus mauritana*. Results are expressed in terms of 4'‐O‐methylgallocatechin (a–d) and *Z. mauritana* proanthocyanidin standard (*zm*‐PAC, e–h). Letters on top of columns represent compact display letters of the Tuckey test. Error bars represent standard deviation.

Figure 3 shows the results for PAC concentration of *Ziziphus mauritiana* samples of green and ripe fruits, leaves, and stems from two harvest locations. PACs were expressed in terms of a reference compound (4'‐O‐methylgallocatechin, or 4‐MGC) and using a standard prepared from the purified extract from the same fruit. The *zm*‐PAC/4‐MGC ratio for all samples was between 4–6 with no specific trend. The *zm*‐PAC was used to represent a more realistic mass value because it contains a similar molecular weight distribution respecting the fruit.

For example, PAC's highest value for leaves was 18 mg 4‐MGC/g (Figure 3c), but the same sample corresponds to 79 mg *zm*‐PAC/g because ber samples contain polymeric proanthocyanidins, not well represented by the 4‐MGC monomer.

Table 3 compares the ber samples contained in this study with several other fruits. PACs in ber samples titer for 1.8–18 mg 4‐MGC/g. Our highest PAC content was greater than the reported for many fruits such as avocado, apricot, figs, raisins, or blackberries. In contrast, ber PAC's content was lower than many fruits, especially from the *Prunus* genus, such as cherry, peach, and nectarine.

**TABLE 3:** Total Proanthocyanidine content in selected fruits.

Fruit	Specie	Country	mg/g DM	Method/standard	Reference
Avocado	*Persea americana*	USA	1.1	HPLC/blueberrry	Wang et al. (2010)
Apricot	*Prunus armeniaca*	Algeria	2.24	Photometric/leucoanthocyanidin	Ouchemoukh et al. (2012)
Apricot	*Prunus armeniaca*	Spain	3.04	HPLC/epicathechin	Redondo et al. (2017)
Figs	*Ficus carica*	Algeria	2.18	Photometric/leucoanthocyanidin	Ouchemoukh et al. (2012)
Raisins	*Vitis vinifera*	Algeria	7.39	Photometric/ leucoanthocyanidin	Ouchemoukh et al. (2012)
Cherry	*Prunus avium*	Spain	10.54	HPLC/epicathechin	Redondo et al. (2017)
Peach	*Prunus persica*	Spain	13.79	HPLC/epicathechin	Redondo et al. (2017)
Nectarine	*Prunus persica*	Spain	59.89	HPLC/epicathechin	(Redondo et al., 2017)
Blackberry	*Rubus adenotrichos*	Costa Rica	2.50–9.26	Photometric/4'‐O‐methyl‐gallocatechin	Araya et al. (2017)
Pear jujube	*Ziziphus jujuba*	China	1.5–5.5	Photometric /Grass‐seed	Wu et al. (2012)
Ber	*Ziziphus mauritana*	Costa Rica	1.8–9.9	Photometric/4'‐O‐methyl‐gallocatechin	This study

Although there are some differences between the results respecting the method and standard utilized, the values of Table 3 provide reference values.

### 3.4 Antioxidant activity

**FIGURE 4:**
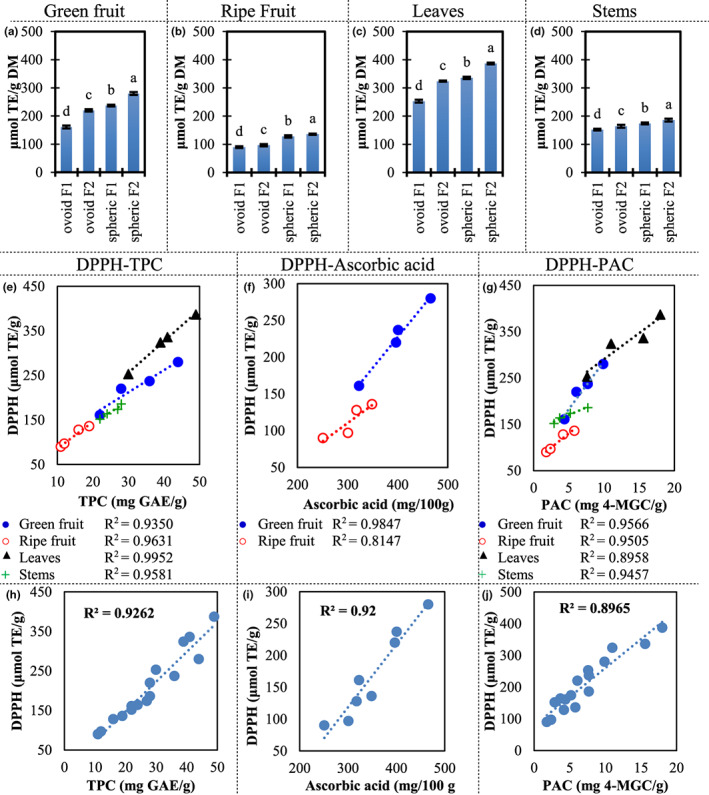
Antioxidant activity determined by the DPPH method and co‐relationship between antioxidant activities and metabolite concentration for two ber cultivars. Antioxidant capacity for (a): green fruits, (b): ripe fruits, (c): leaves, and (d): stems are expressed as Trolox equivalents (TE). Letters on top of columns represent compact display letters of the Tuckey test. Error bars represent standard deviation. Linear regression for DPPH antioxidant activity and: total polyphenolic compounds (TPC), ascorbic acid, and proanthocyanidins (PAC) grouped by plant tissue: (e–g), and all samples together: (h), (i) and (j), respectively.

As mentioned before, the ascorbic acid content was remarkably high in *Z. mauritiana*, respecting many other vitamin C‐rich fruits. Our samples contain high TPC as well. Figure 4a–d shows the antioxidant capacity by measuring DPPH antiradical activity. Our samples showed the antioxidant activity of 90–387 μmol TE/g. Those values were under some berries such as blackberry 551–2151 μmol TE/g (Araya et al., 2017), a well‐known antioxidant‐rich fruit. However, the antioxidant activity was over the reported for some common fruits. For example, antioxidant activity was 2–52 μmol TE/g in apples (Xu et al., 2016), and 0.4–1.0 μmol TE/g in oranges (M'hiri et al., 2017).

To understand the contribution of the different types of antioxidants evaluated in this study (TPC, PAC, and ascorbic acid) to the antioxidant activity, we evaluated the linear regressions of those compounds respecting the TE values for green and ripe fruits, leaves, and stems (Figure 4e–g). Curiously, in our samples, the trends of TPC, ascorbic acid, and PAC were very similar. Also, the *R* ^2^ values for all the linear regressions showed a good fit (0.8147–0.9952). More important, most coefficients of determination are over 0.9. Also, the same behavior is observed when all samples are combined in a single regression (Figure 4h–j).

Previous studies including *Ziziphus mauritiana* and other species of the genus did not show a fit as high as in our experiments (Samirana et al., 2017; Yahia et al., 2020). Also, the phenolic compounds or a subgroup of the phenolic compounds usually show the best fit.

In the conditions included in this study, the biosynthesis and degradation of TPC, PAC, and ascorbic acid seemed to be related or at least occurring in parallel. However, it does not mean we can extrapolate our results to any growth, maturity, ripening, or harvesting conditions.

### 3.5 Antibacterial activity

**FIGURE 5:**
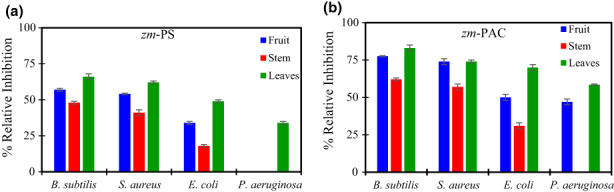
Antibacterial properties of extracts obtained from samples of two cultivars of ber. Results are expressed as a percentage of relative inhibition, using 30 μg chloramphenicol as the positive control. Error bars represent standard deviation.

Finally, we studied the fruit‐derived standards *zm*‐PS and *zm*‐PAC containing the purified fraction of TPC and PAC, respectively, in terms of their capacity of inhibiting bacteria. We included two Gram‐positive and two Gram‐negative bacteria. Figure 5 shows the results of the antibiogram expressed as % Relative Inhibition, respecting chloramphenicol (positive control).

Both standards (*zm*‐PS and *zm*‐PAC) inhibit the two Gram‐positive bacteria included in this study (*B. subtilis* and *S. aureus*). *zm*‐PAC inhibition is stronger than *zm*‐PS, and *zm*‐PAC from fruits and leaves reaches values around 75% of the relative inhibition in the Gram‐positives mentioned above.

Both *zm*‐PS and *zm*‐PAC from either fruit, stems, and leaves inhibit the growth of Gram‐negative *E. coli* and *P. aeruginosa*. The other Gram‐negative evaluated in this work is inhibited by both *zm*‐PS and *zm*‐PAC from leaves and the *zm*‐PAC from the fruit; however, this one is not inhibited by the standards from stems or the *zm*‐PS from the fruit. Therefore, the standards extracted from *Z. mauritiana* have an interesting spectrum of action.

## 4 CONCLUSIONS

There was a dependence on the main antioxidant nutritional quality parameters of ber fruits to the maturity conditions. The ber fruits, which are endemic to Asia, had been previously adapted to live in Costa Rica, and this is the first assessment of nutritional parameters. This fruit is rich in vitamin C and TPC. The concentration of ascorbic acid and total polyphenols was even higher than the one reported in bers grown in other countries such as Thailand, India, Pakistan, and others. From the two cultivars growing in Costa Rica, the spheric cultivar is richer in TPC, and PAC than the ovoid cultivar. Additionally, the farm location makes some differences between the metabolite productivity. There was a high correlationship between all TPC, PAC, and ascorbic acid with the antioxidant activity. The TPC and PACs had an interestingly wide antimicrobial spectrum, being active against *B. subtilis*, *S. aureus*, and *E. coli*, and some of them were against *P. aeruginosa* as well.

## FUNDING INFORMATION

This research has been supported by Universidad Nacional, Heredia, Costa Rica, through the project SIA 89–22 (LAFIT: Phytochemistry laboratory).

## CONFLICT OF INTEREST STATEMENT

The authors declare no competing interests.

## Supporting information


Figure S1.
Figure S2.
Figure S3.

## Data Availability

The data that support the findings of this study are available from the corresponding author upon reasonable request.

## References

[fsn33317-bib-0001] Abalaka, M. , Daniyan, S. , & Mann, A. (2010). Evaluation of the antimicrobial activities of two Ziziphus species (*Ziziphus mauritiana* L. and *Ziziphus spinachristi* L.) on some microbial pathogens. African Journal of Pharmacy and Pharmacology, 4(4), 135–139.

[fsn33317-bib-0002] Araya, M. , Carvajal, Y. , Alvarez, V. , Orozco, R. , & Rodriguez, G. (2017). Polyphenol characterization of three varieties of blackberry fruits (*Rubus adenotrichos*), cultivated in Costa Rica. Journal of Berry Research, 7(2), 97–107.

[fsn33317-bib-0003] Ashraf, A. , Sarfraz, R. A. , Anwar, F. , Shahid, S. A. , & Alkharfy, K. M. (2015). Chemical composition and biological activities of leaves of *Ziziphus mauritiana* L. native to Pakistan. Pakistan Journal of Botanics, 47(1), 367–376.

[fsn33317-bib-0004] Birmingham, A. D. , Esquivel‐Alvarado, D. , Maranan, M. , Krueger, C. G. , & Reed, J. D. (2021). Inter‐laboratory validation of 4‐(dimethylamino) cinnamaldehyde (DMAC) assay using cranberry proanthocyanidin standard for quantification of soluble proanthocyanidins in cranberry foods and dietary supplements, first action official method SM: 2019.06. Journal of AOAC International, 104(1), 216–222. 33251544 10.1093/jaoacint/qsaa084

[fsn33317-bib-0005] Dahiru, D. , Sini, J. , & John‐Africa, L. (2006). Antidiarrhoeal activity of *Ziziphus mauritiana* root extract in rodents. African Journal of Biotechnology, 5(10), 941–945.

[fsn33317-bib-0006] Dahiru, D. , William, E. , & Nadro, M. (2005). Protective effect of *Ziziphus mauritiana* leaf extract on carbon tetrachloride‐induced liver injury. African Journal of Biotechnology, 4(10), 1177–1179.

[fsn33317-bib-0007] Feliciano, R. P. , Shea, M. P. , Shanmuganayagam, D. , Krueger, C. G. , Howell, A. B. , & Reed, J. D. (2012). Comparison of isolated cranberry (*Vaccinium macrocarpon* Ait.) proanthocyanidins to catechin and procyanidins A2 and B2 for use as standards in the 4‐(dimethylamino) cinnamaldehyde assay. Journal of Agricultural Food Chemistry, 60(18), 4578–4585. 22533362 10.1021/jf3007213

[fsn33317-bib-0008] Hasan, S. K. , Kabir, M. R. , Kabir, M. R. , Islam, M. R. , Akhter, M. J. , & Moury, J. Y. (2022). Proximate composition, minerals, phytochemicals, and functional activities of jujube fruits grown in Bangladesh. Journal of Agriculture Food Research, 8, 100302.

[fsn33317-bib-0009] Huang, J. , Heyduck, R. , Richins, R. D. , VanLeeuwen, D. , O'Connell, M. A. , & Yao, S. (2017). Jujube cultivar vitamin C profile and nutrient dynamics during maturation. HortScience, 52(6), 859–867.

[fsn33317-bib-0010] INDER . (2014). Basic characterization of Paquera‐Cóbano‐Lepanto‐Chira territory . Retrieved from: https://www.inder.go.cr/territorio‐peninsular/Caracterizacion‐Paquera‐Cobano‐Lepanto‐Chira.pdf

[fsn33317-bib-0011] Koley, T. K. , Kaur, C. , Nagal, S. , Walia, S. , & Jaggi, S. (2016). Antioxidant activity and phenolic content in genotypes of Indian jujube (*Zizyphus mauritiana* Lamk.). Arabian Journal of Chemistry, 9, S1044–S1052.

[fsn33317-bib-0012] Koley, T. K. , Walia, S. , Nath, P. , Awasthi, O. , & Kaur, C. (2011). Nutraceutical composition of *Zizyphus mauritiana* Lamk (Indian ber): Effect of enzyme‐assisted processing. International Journal of Food Sciences Nutrition, 62(3), 276–279. 21091292 10.3109/09637486.2010.526930

[fsn33317-bib-0013] MAG‐INTA . (2022). la fruticultura tropical una opcion para el desarrollo agropecuario en costa rica . Retrieved from http://www.mag.go.cr/bibliotecavirtual/E71‐8445.pdf

[fsn33317-bib-0014] Mesén‐Mora, L. D. , Carvajal‐Miranda, Y. , Álvarez‐Valverde, V. , & Rodríguez‐Rodríguez, G. (2019). Bioprospecting study, antibiotic and antioxidant activity of the santol's fruit (*Sandoricum koetjape*). Uniciencia, 33(1), 75–82.

[fsn33317-bib-0015] M'hiri, N. , Irina, I. , Cédric, P. , Ghoul, M. , & Boudhrioua, N. (2017). Antioxidants of maltease orange peel: Comparative investigation of the efficiency of four extraction methods. Applied Pharmaceutical Sciences, 7(11), 126–135.

[fsn33317-bib-0016] Ouchemoukh, S. , Hachoud, S. , Boudraham, H. , Mokrani, A. , & Louaileche, H. (2012). Antioxidant activities of some dried fruits consumed in Algeria. LWT‐Food Science Technology, 49(2), 329–332.

[fsn33317-bib-0017] Prakash, O. , Usmani, S. , Singh, R. , Singh, N. , Gupta, A. , & Ved, A. (2021). A panoramic view on phytochemical, nutritional, and therapeutic attributes of *Ziziphus mauritiana* lam.: A comprehensive review. Phytotherapy Research, 35(1), 63–77. 32633009 10.1002/ptr.6769

[fsn33317-bib-0018] Redondo, D. , Arias, E. , Oria, R. , & Venturini, M. E. (2017). Thinned stone fruits are a source of polyphenols and antioxidant compounds. Journal of the Science of Food Agriculture, 97(3), 902–910. 27219821 10.1002/jsfa.7813

[fsn33317-bib-0019] Samirana, P. O. , Susidarti, R. A. , & Rohman, A. (2017). Isolation and 2, 2′‐diphenyl‐1‐picrylhydrazyl radical scavenging activity of active compound from jujube tree (*Zizyphus mauritiana* Auct. Non Lamk.). International Journal of Food Properties, 20(sup2), 1523–1529.

[fsn33317-bib-0020] San, A. M. M. , Thongpraditchote, S. , Sithisarn, P. , & Gritsanapan, W. (2013). Total phenolics and total flavonoids contents and hypnotic effect in mice of *Ziziphus mauritiana* lam. Seed extract. Evidence‐Based Complementary Alternative Medicine, 2013, 835854. 23861716 10.1155/2013/835854 PMC3687505

[fsn33317-bib-0021] Satpathy, L. , Pradhan, N. , Dash, D. , & Priyadarshini, P. (2021). Quantitative determination of vitamin C concentration of common edible food sources by redox titration using iodine solution. Letters in Applied NanoBioScience, 10(3), 23612369.

[fsn33317-bib-0022] Shyla, B. , & Nagendrappa, G. (2013). Redox spectrophotometric method involving electrolytically generated manganese (III) sulphate with diphenylamine for the determination of ascorbic acid present in the samples of various fruits, commercial juices and sprouted food grains. Food Chemistry, 138(2–3), 2036–2042. 23411340 10.1016/j.foodchem.2012.11.076

[fsn33317-bib-0023] Syedd‐León, R. , Orozco, R. , Álvarez, V. , Carvajal, Y. , & Rodríguez, G. (2020). Chemical and antioxidant charaterization of native corn germplasm from two regions of Costa Rica: A conservation approach. International Journal of Food Science 2020, 2439541. 32090061 10.1155/2020/2439541 PMC7014556

[fsn33317-bib-0024] Valente, A. , Albuquerque, T. G. , Sanches‐Silva, A. , & Costa, H. S. (2011). Ascorbic acid content in exotic fruits: A contribution to produce quality data for food composition databases. Food Research International, 44(7), 2237–2242.

[fsn33317-bib-0025] Wang, W. , Bostic, T. R. , & Gu, L. (2010). Antioxidant capacities, procyanidins and pigments in avocados of different strains and cultivars. Food Chemistry, 122(4), 1193–1198.

[fsn33317-bib-0026] Wojdyło, A. , Carbonell‐Barrachina, Á. A. , Legua, P. , & Hernández, F. (2016). Phenolic composition, ascorbic acid content, and antioxidant capacity of Spanish jujube (*Ziziphus jujube* mill.) fruits. Food Chemistry, 201, 307–314. 26868581 10.1016/j.foodchem.2016.01.090

[fsn33317-bib-0027] Wu, C.‐S. , Gao, Q.‐H. , Guo, X.‐D. , Yu, J.‐G. , & Wang, M. (2012). Effect of ripening stage on physicochemical properties and antioxidant profiles of a promising table fruit ‘pear‐jujube’(*Zizyphus jujuba* mill.). Scientia Horticulturae, 148, 177–184.

[fsn33317-bib-0028] Xu, Y. , Fan, M. , Ran, J. , Zhang, T. , Sun, H. , Dong, M. , Zhang, Z. , & Zheng, H. (2016). Variation in phenolic compounds and antioxidant activity in apple seeds of seven cultivars. Saudi Journal of Biological Sciences, 23(3), 379–388. 27081364 10.1016/j.sjbs.2015.04.002 PMC4818338

[fsn33317-bib-0029] Yahia, Y. , Benabderrahim, M. A. , Tlili, N. , Bagues, M. , & Nagaz, K. (2020). Bioactive compounds, antioxidant and antimicrobial activities of extracts from different plant parts of two *Ziziphus* mill. Species. PLoS One, 15(5), e0232599. 32428000 10.1371/journal.pone.0232599 PMC7236975

[fsn33317-bib-0030] Zamora, N. , Hammel, B. , Grayum, M. , Herrera, C. , & Zamora, N. (2010). Manual de plantas de Costa Rica. Monographs in Systematic Botany from the Missouri Botanical Garden, 93, 101–169.

[fsn33317-bib-0031] Zhang, Q. , Wang, L. , Wang, Z. , Liu, Z. , Zhao, Z. , Zhou, G. , Liu, M. , & Liu, P. (2020). Variations of the nutritional composition of jujube fruit (*Ziziphus jujuba* mill.) during maturation stages. International Journal of Food Properties, 23(1), 1066–1081.

[fsn33317-bib-0032] Zozio, S. , Servent, A. , Cazal, G. , Mbéguié‐A‐Mbéguié, D. , Ravion, S. , Pallet, D. , & Abel, H. (2014). Changes in antioxidant activity during the ripening of jujube (*Ziziphus mauritiana* Lamk). Food Chemistry, 150, 448–456. 24360474 10.1016/j.foodchem.2013.11.022

[fsn33317-bib-0033] Zozio, S. , Servent, A. , Hubert, O. , Hiol, A. , Pallet, D. , & Mbéguié‐A‐Mbéguié, D. (2014). Physicochemical and biochemical characterization of ripening in jujube (*Ziziphus mauritiana* Lamk) fruits from two accessions grown in Guadeloupe. Scientia Horticulturae, 175, 290–297.

