# Supplementary material for: Antioxidant and antimicrobial activity of two Costa Rican cultivars of ber (Ziziphus mauritiana): An underexploited crop in the American tropic
Source: Food Sci Nutr. 2023 Mar 17;11(6):3320–8. doi: 10.1002/fsn3.3317 (PMC10261771; doi:10.1002/fsn3.3317)
Supplement: Supplementary file 1 — Figure S1. Figure S2. Figure S3. [file FSN3-11-3320-s001.docx]

Supplementary material

Antioxidant and antimicrobial activity of two Costa Rican cultivars of ber (*Ziziphus mauritiana*): an underexploited crop in the American tropic.

**Eric Cubero-Román**, **Yendry Carvajal-Miranda**, **Gerardo Rodríguez**, **Victor Álvarez-Valverde**, **Pablo Jiménez-Bonilla**

*Corresponding author

Pablo Jiménez Bonilla,

School of Chemistry

Universidad Nacional (UNA)

New Industrial Processes Bldg,

Omar Dengo Campus, Heredia, 40101 Costa Rica

Tel: 506-2277-3555

E-mail: [pablo.jimenez.bonilla@una.cr](mailto:pablo.jimenez.bonilla@una.cr)

Supplementary figures

| (A) |  | |  | |  |  |
| --- | --- | --- | --- | --- | --- | --- |
| 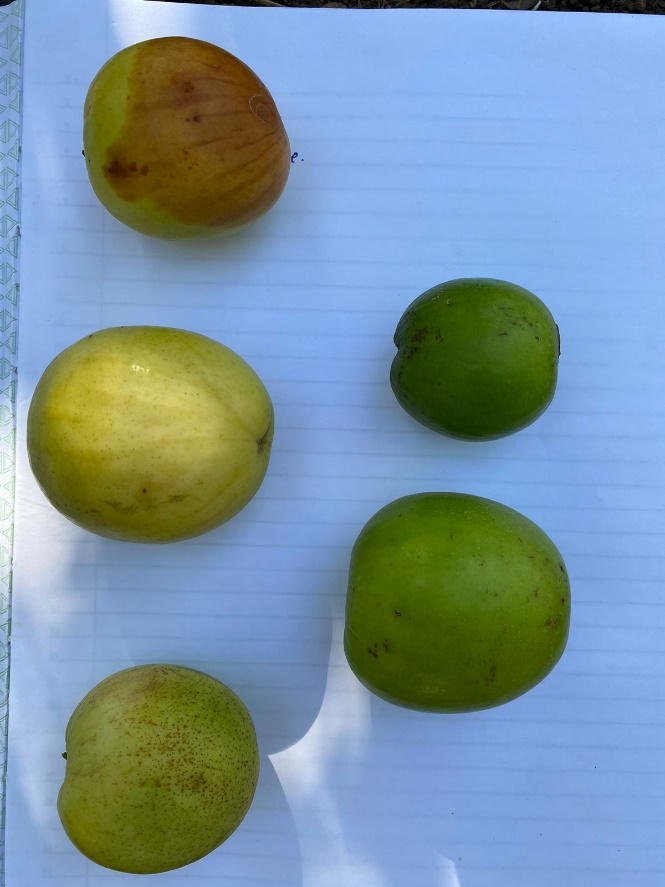 | 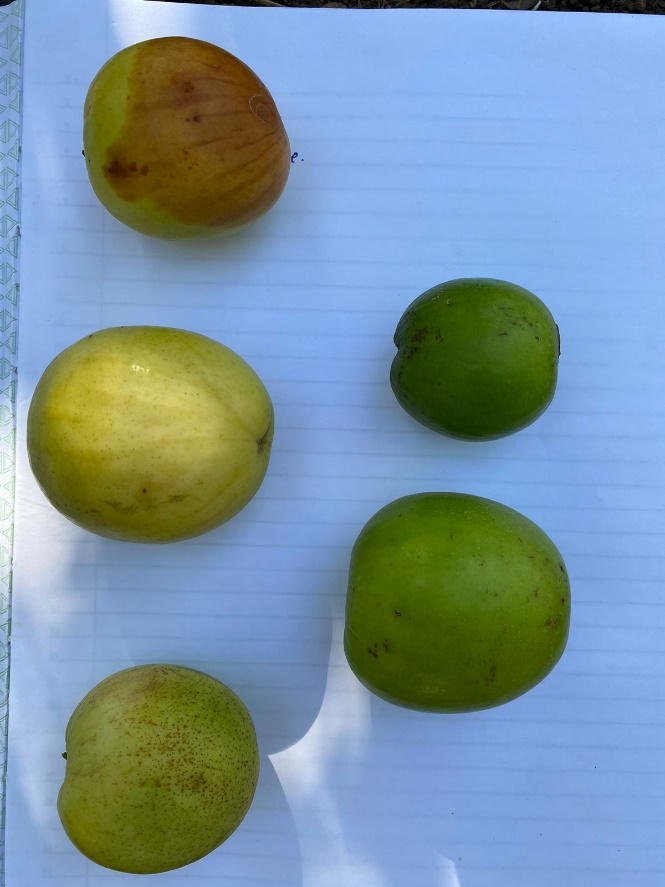 | | 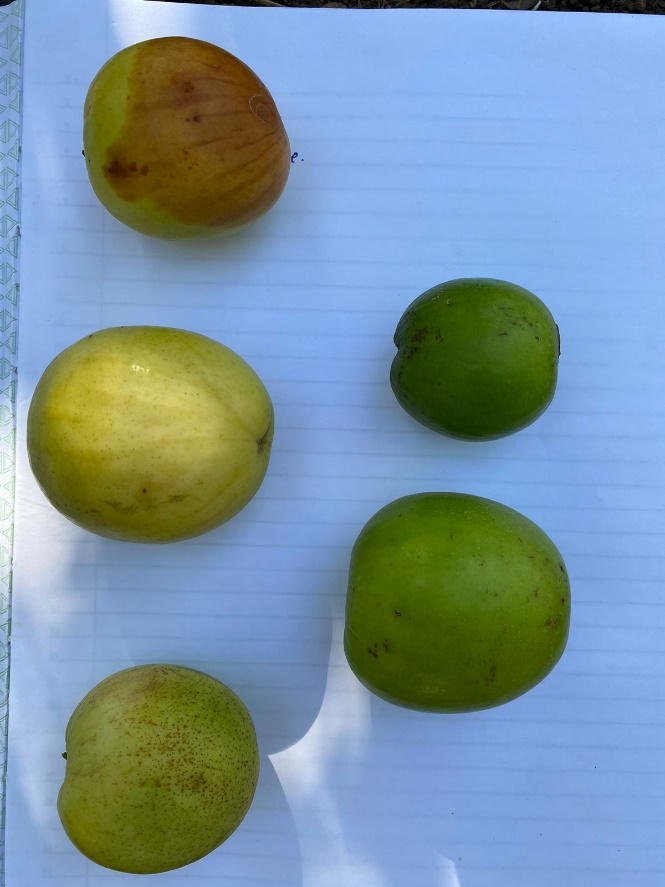 | | 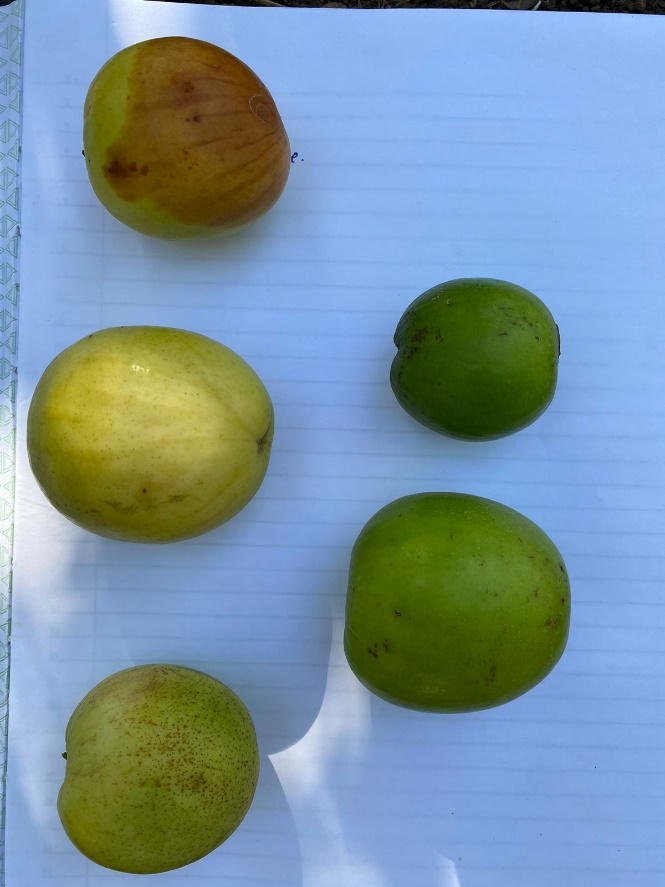 | 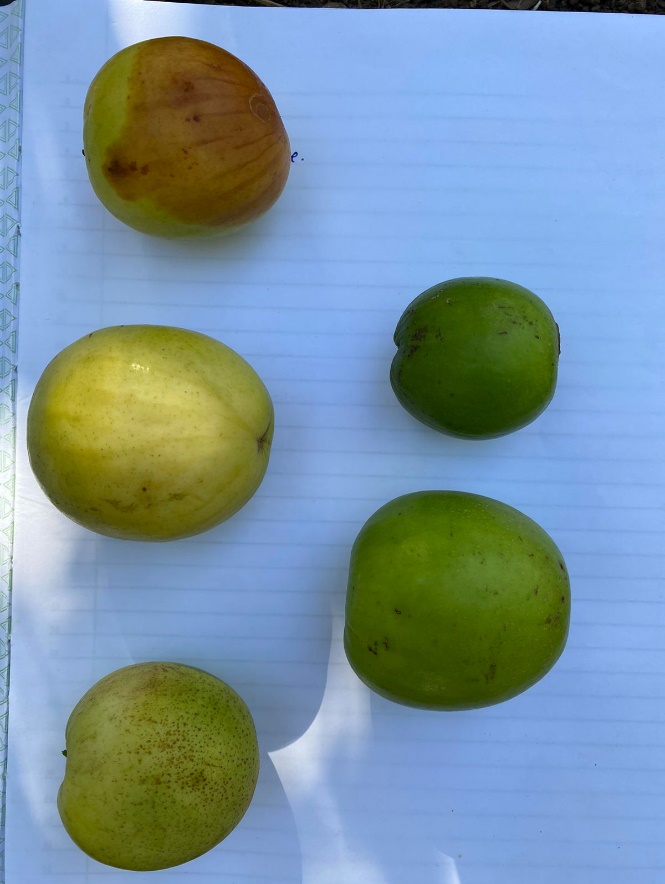 |
| Stage 0 | Stage 1 (green) | | Stage 2 (ripe) | | Stage 3 | Stage 4 |
| (B) |  | |  | (C) | |  |
| 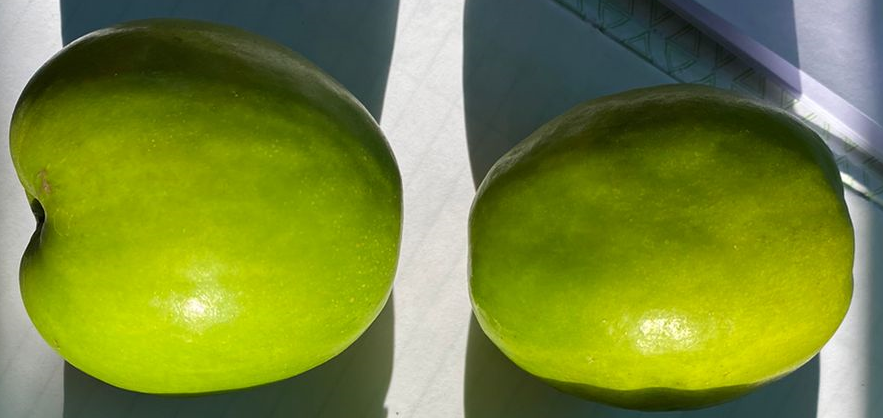 | | | | 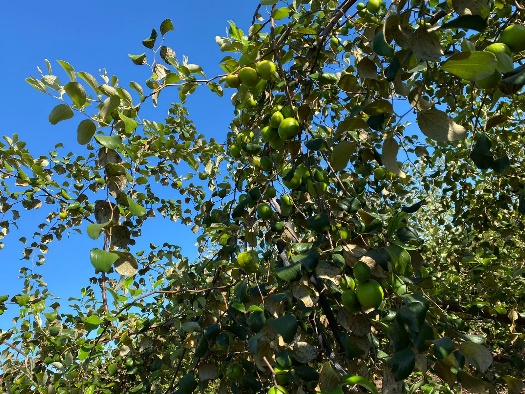 | | |
| Round-shaped | | Ovoid-shaped | |  |  |  |

Figure S1. *Ziziphus mauritiana* fruits. (A) Maturity stages. (B) Cultivars. (C) fruits in trees.

Fruits needs to ripe in the trees in order to be harvested. Stage 1 and 2 are those considered in this study. Fruits grow during dry season in Costa Rica, and are consumed locally, mainly. Ovoid cultivar is identified as “sweeter” than round cultivar (a little bit bitter than ovoid).

Figure S2. Total polyphenolic content in fruits, leaves and steams extracted with 4 different solvents, as a preliminary test for the identification of the best extraction solvent.

Figure S3. Determination of the optimal number of extraction cycles of *Z.* *mauritiana*, yellow, green, and pink bars represent fruits, leaves, and stems, respectively. Letters in top of bars represent groups for Tukey´s test *(P≤0.05).*
